# Entomological surveillance of Chagas disease in the East of Minas Gerais region, Brazil.

**DOI:** 10.1590/0037-8682-0065-2022

**Published:** 2022-09-19

**Authors:** Mariana de Almeida Rosa Rezende, Marta de Lana, Liléia Diotaiuti, Girley Francisco Machado-de-Assis

**Affiliations:** 1 Universidade Federal de Juiz de Fora, Campus Governador Valadares, Programa de Pós-Graduação em Ciências Aplicadas à Saúde, Governador Valadares, MG, Brasil.; 2 Universidade Federal de Ouro Preto, Programa de Pós-Graduação em Ciências Biológicas e Programa de Pós-Graduação em Ciências Farmacêuticas, Ouro Preto, MG, Brasil.; 3 Centro de Pesquisas René Rachou, Laboratório de Triatomíneos e Epidemiologia da Doença de Chagas, Belo Horizonte, MG, Brasil.; 4 Universidade Federal de Juiz de Fora, Campus Governador Valadares, Programa de Pós-Graduação em Ciências Aplicadas à Saúde, Laboratório de Parasitologia, Governador Valadares, MG, Brasil.

**Keywords:** Triatomine, Vector control, Trypanosoma cruzi, T. vitticeps, Entomological surveillance

## Abstract

**Background:**

After decentralizing the actions of the Chagas Disease Control Program (CDCP) in Brazil, municipalities were now responsible for control measures against this endemic, supervised by the Regional Health Superintendencies (RHS). We aimed to evaluate the recent entomological surveillance of Chagas disease in the Regional Health Superintendence of Governador Valadares (RHS/GV) from 2014 to 2019.

**Methods:**

Triatomines captured by residents during entomological surveillance were sent to the reference laboratory, where the species and evolutionary stages were identified, place of capture, and presence of *Trypanosoma cruzi*. A database was created, and the following were calculated: the rate of infection by *T. cruzi* (overall rate and rate by species), monthly seasonality, spatial distribution of species, number of captures, and infected triatomines/health microregions.

**Results:**

We identified 1,708 insects; 1,506 (88.2%) were triatomines, most were adult instars (n=1,469), and few were nymphs (n=37). The identified species were *Triatoma vitticeps*, *Panstrongylus megistus*, *Panstrongylus diasi*, *Rhodnius neglectus,* and *Panstrongylus geniculatus*. The first three were most frequently captured and distributed throughout the study area. Most bugs were captured intradomicile (72.5%), mainly in the second semester, between September and November, with an average infection rate of 41.5% (predominantly *T. vitticeps*, 49.2%). All municipalities sent triatomines, especially in the microregions of Governador Valadares.

**Conclusions:**

These data reinforce the need and importance of improving Chagas disease control measures in the region to establish active and participatory entomological surveillance.

## INTRODUCTION

It is estimated that 5,742,167 people are infected with *Trypanosoma cruzi* worldwide, and 1.5 million of them are Brazilians^1^. In Brazil, a seroepidemiological survey conducted in rural areas revealed an overall prevalence of 4.2% positivity for *T. cruzi* infection^2^. Minas Gerais had the highest prevalence rate, representing 8.8% of the population. In parallel, an entomological survey carried out between 1975 and 1983 by direct search for infestation in houses, revealed areas of wide occurrence of domiciled triatomines^3^. The data from these two surveys were fundamental for determining the priority for controlling the transmission of Chagas disease. Attacking the vector is the most viable strategy, with extensive use of insecticides with residual action in infested locations^4^. Based on these initial surveys, areas at risk of vector transmission were defined and should have priority for control interventions, including 3,372 municipalities. Triatomine species responsible for domestic transmission were also identified, in order of importance: *Triatoma infestans*, *Panstrongylus megistus*, *T. brasiliensis*, *T. sordida* and *T. pseudomaculata*. Eastern Minas Gerais was not included in this priority area, despite evidence of human infection being confirmed in several municipalities, with prevalence rates varying between 0.2 and 12.4%^2^. Decades after these initiatives, the determinants allowing the occurrence of Chagas disease in the area corresponding to RHS/GV, Minas Gerais, remain unrecognized. Some municipalities have implemented surveillance activities for the presence of triatomines in homes; however, neither the data nor the methodology used were systematically evaluated. To elucidate this, the study aimed to collect information on the occurrence and infestation of households by triatomines in this region.

## METHODS

**FIGURE 1:**
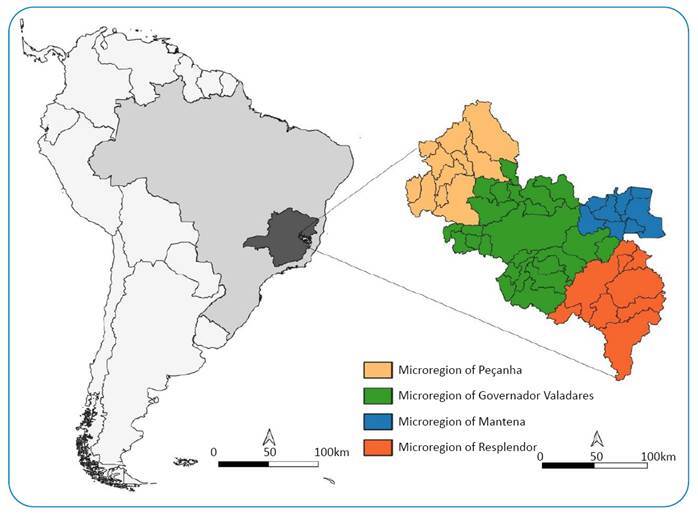
Localization and division of Governador Valadares Regional Health Superintendence in microregion.

The study was conducted in municipalities under the jurisdiction of RHS/GV in eastern Minas Gerais, Brazil. This area is composed of 51 municipalities divided into 4 health microregions: Peçanha, Governador Valadares, Mantena, and Resplendor (Figure 1). It is a region with a significantly degraded Atlantic forest and high temperature.

The insects captured by the residents during passive surveillance from January 2014 to December 2019 were sent to the reference laboratory at RHS/GV. The insects were identified according to Alevi et al.^5^. *T. cruzi* infection in the digestive tract of triatomines was determined by analyzing the intestinal contents. Pressure was applied in the terminal part of the intestine diluted in PBS under a binocular microscope for analysis.

The notification forms were analyzed according to the place of capture, date, and municipality where the captures were performed. This information was compiled using Microsoft Office Excel 2016. From this database, the natural infection rate of these triatomines was calculated (number of triatomines infected by *T. cruzi*/number of triatomines examined × 100)^6^, and the monthly seasonality of catches was determined. The spatial distribution of species was determined using Quantum GIS3.10 program and the free cartography base of IBGE. The research was approved by the Research Ethics Committee of the UFJF (number CAAE:34754520.5.0000.5147). Usage of data from the RHS/GV was authorized by the regional superintendent via a cooperation agreement signed with the Federal University of Juiz de Fora, campus Governador Valadares. 

## RESULTS

From 2014 to 2019, residents of the municipalities captured 1,708 insects, where1,506 were triatomines, 97.5% were adults, and 2.5% were nymphs. We identified 1,490 triatomines, predominantly *Triatoma vitticeps*, followed by *Panstrongylus megistus, Panstrongylus diasi, Rhodnius neglectus*, and *Panstrongylus geniculatus*. *T. cruzi* infected 41.5% of triatomines, with an even higher infection rate for *T. vitticeps* (49.2%). Aside from *P. geniculatus*, all species were mostly captured (72.5%) within domiciles (Table 1).

**TABLE 1:** Numbers of captured triatomines, classified by species, place of capture, examination, and infection with *Trypanosoma cruzi*, sent for laboratory analysis by municipalities under the jurisdiction of the Governador Valadares Regional Health Superintendence, between 2014 and 2019.

Species	Place of capture			Total (%)	Examined	Infected (%)
	Intra	Peri	NI			
*T. vitticeps*	737	194	20	951 (63.8%)	824	405 (49.2%)
*P. megistus*	184	93	6	283 (19.1%)	233	88 (37.8%)
*P. diasi*	152	87	5	244 (16.4%)	177	17 (9.6%)
*R. neglectus*	8	3	0	11 (0.7%)	8	5 (62.5%)
*P. geniculatus*	0	1	0	1 (0.1%)	0	0
**Total**	**1,081 (72.5%)**	**378 (25.4%)**	**31 (2.1%)**	**1,490 (100%)**	**1,242 (83,4%)**	**515 (41.5%)**

Intra: Intradomicile; Peri: Peridomicile; NI: Not identified.

During this study, triatomines were captured in all municipalities in the study area with an average annual participation of 39 municipalities. All species were captured mainly during the second half of the year, (September-November). Although captures were carried out in all municipalities, a considerable number of specimens were identified in the following municipalities: Conselheiro Pena (n=144), Tarumirim (n=108), Governador Valadares (n=102), Sobralia (n=97), Capitão Andrade (n=88), José Raydan (n=86), Itanhomi (n=78), Água Boa (n=70), and Aimorés (n=63) (Table 2).

**TABLE 2:** Number of triatomines captured, examined, and infected by *Trypanosoma cruzi* and natural infection rate in the municipalities registered by health microregion, between 2014 and 2019.

Municipalities	Captured	Examined	Infected	Natural infection rate
**Microregion of Governador Valadares**				
Tarumirim	108	95	49	51.6%
Governador Valadares	102	85	29	34.1%
Sobrália	97	89	47	52.8%
Capitão Andrade	88	76	44	57.9%
Itanhomi	78	65	36	55.4%
São Geraldo do Baixio	47	42	16	38.1%
Frei Inocêncio	39	35	17	48.6%
São José da Safira	32	24	9	37.5%
Galileia	26	23	8	34.8%
Fernandes Tourinho	22	18	3	16.7%
Engenheiro Caldas	16	11	5	45.5%
Tumiritinga	16	10	2	20%
São Geraldo da Piedade	15	13	8	61.5%
Virgolândia	14	10	7	70%
Nacip Raydan	12	4	0	0%
Gonzaga	10	5	4	80.0%
Alpercata	9	7	3	42.9%
Coroaci	6	4	1	25%
Divinolândia de Minas	4	4	3	75%
Mathias Lobato	4	3	0	0%
Jampruca	3	3	1	33.3%
Santa Efigênia de Minas	3	1	1	100%
Sardoá	2	2	1	50%
Marilac	2	1	1	100%
Total	755	630	295	46.8%
**Microregion of Resplendor**				
Conselheiro Pena	144	119	65	54.6%
Aimorés	63	43	13	30.2%
Resplendor	30	29	6	20.7%
Itueta	27	17	3	17.6%
Goiabeira	22	18	6	33.3%
Santa Rita do Itueto	18	15	7	46.7%
Alvarenga	17	14	5	35.7%
Cuparaque	8	8	3	37.5%
Total	329	263	108	41.1%
**Microregion of Peçanha**				
José Raydan	86	82	31	37.8%
Água Boa	70	58	11	19%
São Sebastião do Maranhão	38	35	3	8.6%
São Pedro do Suaçui	32	26	16	61.5%
São João Evangelista	25	18	7	38.9%
Peçanha	22	17	9	52.9%
Paulistas	16	10	6	60%
São José Jacuri	15	12	3	25%
Santa Maria do Suaçui	15	9	0	0%
Canta Galo	5	4	1	25%
Frei Lagonegro	2	1	1	100%
Total	326	272	88	32.4%
**Microregion of Mantena**				
Mendes Pimentel	26	22	7	31.8%
Divino das Laranjeiras	20	19	3	15.8%
São Felix de Minas	15	13	8	61.5%
Central de Minas	12	8	0	0%
Itabirinha de Mantena	10	8	5	62.5%
Mantena	9	5	0	0%
São João do Manteninha	3	1	1	100%
Nova Belém	1	1	0	0%
Total	96	77	24	31.2%
Grand total	1,506	1,242	515	41.5%

Only 6 of the 51 municipalities studied did not capture infected triatomines. The municipalities that sent the most *T. cruzi­­*-infected triatomines were: Conselheiro Pena (n=65), Tarumirim (n=49), Sobralia (n=47), Capitão Andrade (n=44), Itanhomi (n=36), José Raydan (n=31), and Governador Valadares (n=29) (Table 2).

**FIGURE 2:**
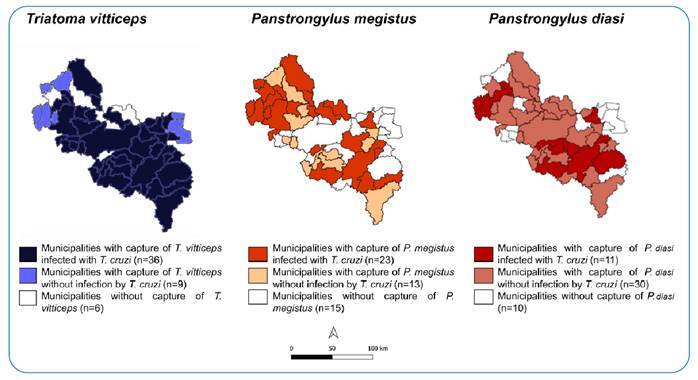
Spacial distribution of municipalities with capture of triatomines according to species: *Triatoma vitticeps; Panstrongylus megistus* and *Panstrongylus diasi*, infected or not between 2014 and 2019.

*T. vitticeps* had a broader dispersion (45 municipalities) and wider *T. cruzi* infection (n=36). *P. megistus* was captured in 36 municipalities, where 23 were infected, whereas *P. diasi* was captured in 41 municipalities, where 11 were infected. *R. neglectus* was captured only in Governador Valadares, with specimens infected by *T. cruzi*. *P. geniculatus* was captured once in Itanhomi and was not examined for infection (Figure 2).

The microregion of Governador Valadares is notable because besides having a more significant number of municipalities under the jurisdiction of the RHS (n=24)-it sent the largest number of triatomine specimens (n=755) and presented a natural infection rate of 46.8%. These captures were mainly concentrated in the municipalities of Tarumirim (n=108), Governador Valadares (n=102), Sobrália (n=97), Capitão Andrade (n=88), and Itanhomi (n=78), which also sent the highest number of infected specimens to the RHS (Table 2).

The microregion of Resplendor sent 329 specimens, captured mainly in the municipalities of Conselheiro Pena (n=144) and Aimorés (n=63), and had a natural infection rate of 41.1%. The microregion of Peçanha submitted 326 triatomine specimens, most of which were captured in José Raydan (n=86) and Água Boa (n=70). Infected specimens were mainly caught in José Raydan (n=31) and São Pedro do Suaçui (n=16), with a natural infection rate of 32.4%. The microregion of Mantena had the smallest number of specimens (n=96) and the lowest natural infection rate (31.2%) (Table 3).

In the microregions of Governador Valadares, Resplendor, and Mantena, the species captured was predominantly *T. vitticeps*, followed by *P. diasi*, and *P. megistus*. Captures were mostly intradomicile, and most of the *T. cruzi*-infected insects were concentrated (Table 3). The triatomines captured in the microregion of Peçanha were predominantly those of *P. megistus*, followed by *T. vitticeps* and *P. diasi*, and infected specimens were mostly captured intradomicile (Table 3).

**TABLE 3:** Distribution of triatomines species captured, examined, infected by *Trypanosoma cruzi* and place of capture registered by healths microregions, between 2014 and 2019.

Species	Captured	Examined	Infected (%)	Place of capture			Infected triatomines	
				Intra	Peri	NI	Intra	Peri
**Microregion of Governador Valadares**								
*T. vitticeps*	568	500	269 (53.8%)	434	121	13	218	47
*P. diasi*	127	90	6 (6.7%)	81	43	3	2	3
*P. megistus*	41	32	15 (46.9%)	31	10	0	9	6
*R. neglectus*	11	8	5 (62.5%)	8	3	0	2	3
*P. geniculatus*	1	0	0 (0%)	0	1	0	0	0
Total	748	630	295 (46.8%)	554	178	16	231	59
**Microregion of Resplendor**								
*T. vitticeps*	241	198	99 (50%)	205	30	6	86	12
*P. diasi*	76	57	5 (8.8%)	43	32	1	3	2
*P. megistus*	9	8	4 (50%)	8	1	0	4	0
Total	326	263	108 (41.1%)	256	63	7	93	14
**Microregion of Peçanha**								
*P. megistus*	229	190	67 (35.3%)	141	82	6	48	17
*T. vitticeps*	73	68	16 (23.5%)	34	39	0	13	3
*P. diasi*	18	14	5 (35.7%)	14	3	1	3	1
Total	320	272	88 (32.4%)	189	124	7	64	21
**Microregion of Mantena**								
*T. vitticeps*	69	58	21 (36.2%)	64	4	1	21	0
*P. diasi*	23	16	1 (6.3%)	14	9	0	1	0
*P. megistus*	4	3	2 (66.7%)	4	0	0	2	0
Total	96	77	24 (31.2%)	82	13	1	24	0
	**1,490**	**1,242**	**515 (41.5%)**	**1,081**	**378**	**31**	**412**	**94**

Intra: Intradomicile. Peri: Peridomicile. NI: Not identified.

## DISCUSSION

The capture of insects by the general population reflects the residents’ ability to recognize triatomines capable of transmitting *T. cruzi*, as observed by Vilella et al.^7^ for other regions of Minas Gerais. Between 2014 and 2019, municipalities under the jurisdiction of RHS/GV distinguished triatomines from other insects in 88.2% of the specimens; thus, revealing that residents of the household units in this region are aware of CD vectors. Of the insects notified, 11.8% were phytophages or predators. This also reinforces the need for constant awareness among the population regarding the identification of triatomines, as it is the key to the occurrence of infestation notification. There was a predominance of adult capture (97.5%), revealing the capacity of these vectors to invade houses possibly attracted by light sources^8^ ^,^ ^9^. This is a frequent finding in areas under entomological surveillance^7^. Nymphs are mostly captured when trained personnel perform an active search. It is important to emphasize that the presence of nymphs indicates colonization of the species, either in the household or peridomicile^10^. Detecting nymphs in the household also demonstrates their adaptation to the environment, which may increase contact between the insect and residents.

Captures in the study area were predominantly intradomicile (72.5%), resembling the capture profiles in the macroregions of Minas Gerais of Diamantina^11^ and Divinópolis^12^ and of the Espírito Santo state^13^. In the Itanhomi municipality-located in the Rio Doce Valley and part of the health region assessed in this study-Souza et al.^14^ demonstrated an intense movement of triatomines among the wild, peridomiciliary, and domestic environments, showing high rates of natural infection. 

Of the triatomines reported, 83.4% were examined for *T. cruzi* infection, reinforcing considerable conservation of the triatomines sent for examination. This high percentage of captured and examined insects reflects the timely execution of control actions, aiding the identification and analysis of almost all insects captured by the residents. 

High rates of natural infection by *T. cruzi* have also been reported in regions with similar landscapes, such as Espírito Santo^13^ and Rio de Janeiro^15^ ^,^ ^16^. In contrast, the rates varied between 1.3% and 8.3% in the Midwest region of Minas Gerais^7^ ^,^ ^12^. High rates of triatomine infection increase the chances of vector transmission in cases of vector-human contact; therefore, surveillance should be reinforced so that subsequent cases can be identified immediately and referred for diagnosis and treatment. 

The triatomines identified were *T. vitticeps, P. megistus, P. diasi, R. neglectus,* and *P. geniculatus*, with the first three being predominant. This is the first study reporting the occurrence of *P. diasi, R. neglectus,* and *P. geniculatus* in the studied region. *T. vitticeps* was most commonly captured by residents in the urban area of Diamantina, Minas Gerais^11^_,_ and the Espírito Santo state^13^; areas neighboring the macroregion of Governador Valadares. In this study, we found a natural infection rate of 49.2% for *T. vitticeps*, lower than that found in the Itanhomi municipality^14^ and states of Espírito Santo^13^ and Rio de Janeiro^15^ ^,^ ^16^. Our findings demonstrate the continuity of *T.vitticeps* occurrence between these regions.

*P. megistus* is widely distributed in Brazil, from the northeast to the south^8^. In Minas Gerais, this species also has a wide dispersal area and is considered the most important triatomine in this state^17^. In the health macroregion of Governador Valadares, *P. megistus* was the second most common species, accounting for 18.9% of specimens, with a *T. cruzi* infection rate of 37.8%. The predominance of intradomicile captures, consistent with the findings of the present study, was also reported in other studies, wherein captures were also performed by passive search^11^ ^,^ ^12^. Historical data in the past indicate that the region studied had a considerable rate of natural infection by *T. cruzi* among vector species^18^. Other Minas Gerais and São Paulo state regions have lower natural infection rates for *P. megistus* ^19^ ^,^ ^20^. 

The third most reported species was *P. diasi*, which differs from other regions of Minas Gerais, such as Triângulo Mineiro and Alto Parnaíba. Here, triatomines of this species represent 0.8% of the specimens with none of them being infected^20^. In the Midwest region of Minas Gerais, *P. diasi* representation was even lower^7^, as in Uberlândia, Minas Gerais. The high number of *P. diasi* captured intradomicile, with a natural infection rate of 9.6%, makes this study region notable because of this species in the state. 

For *R. neglectus*, despite the small sample size of 11 insects, the infection of 5 specimens was relevant. Silveira et al.^3^ reported the capture of said species in the Control Program of Chagas Disease in 1975-1983 in 103 municipalities of Minas Gerais state, which did not include the municipalities studied. In the present study, *R. neglectus* was exclusively captured in the Governador Valadares municipality while *P. geniculatus* was only captured in the Itanhomi municipality. Other regions of Minas Gerais have a wide dispersion of this species, but with a low frequency^20^. 

Considering the monthly frequency of capture, a predominance of triatomines was observed in the second semester, especially between September and November. Therefore, the triatomine species found in the region east of Minas Gerais were captured predominantly between spring and summer.

Triatomine capture occurred at least once between 2014 and 2019, with an average of 39 municipalities annually. The participation of municipalities in notifying insects for control actions is frequent, demonstrating good adherence to entomological monitoring.

The spatial distribution revealed a wide dispersion of *T. vitticeps, P. megistus*, and *P. diasi*, with specimens infected by *T. cruzi* in all species. The data presented here show the predominance of *T. vitticeps* in the microregions bordering the state of Espírito Santo, where this species is also predominant^13^. *P. megistus* is the predominant species in the microregion of Peçanha, which borders municipalities belonging to the health macroregion of Diamantina, where *P. megistus* is predominantly captured^21^. This geographical proximity between municipalities and similar environmental features favor the proliferation of certain species to the detriment of others. 

Considering the number of specimens captured and triatomines infected, the municipalities of Conselheiro Pena, Tarumirim, Governador Valadares, Sobrália, Capitão Andrade, José Raydan, and Itanhomi were noteworthy. Of these municipalities, 5 belong to the microregion of Governador Valadares, making this the area with the most significant capture and highest average natural infection rate (46.8%); that is, a region that demands greater attention in the execution of its activities, considering the risk of vectorial transmission of *T. cruzi*. 

In conclusion, the data presented here demonstrate the diversity of triatomine species, a constant presence invading the intradomicile environment, and a considerable rate of infection by *T. cruzi* in the municipalities studied. Therefore, actions must be taken to improve entomological surveillance as well as strengthen and enhance active and participative surveillance.
